# A single-blinded, single-centre, controlled study in healthy adult smokers to identify the effects of a reduced toxicant prototype cigarette on biomarkers of exposure and of biological effect versus commercial cigarettes

**DOI:** 10.1186/1471-2458-13-690

**Published:** 2013-07-29

**Authors:** Christopher J Shepperd, Nik Newland, Alison Eldridge, Don Graff, Ingo Meyer

**Affiliations:** 1 British American Tobacco, Group Research and Development, Regents Park Road, Southampton SO15 8TL, UK; 2 Celerion, 621 Rose St, Lincoln, NE 68502, USA; 3 MPS Hamburg GmbH, Kieler Strasse 99-105, Hamburg 22769, Germany

**Keywords:** Biomarker of exposure, Biomarker of biological effect, Tobacco smoke toxicants, Reduced toxicant prototype cigarettes, Potential reduced-exposure product, PREP, Modified risk tobacco product, MRTP, Smoking

## Abstract

**Background:**

Despite universal acceptance that smoking is harmful, a substantial number of adults continue to smoke. The development of potential reduced exposure products (more recently termed modified risk tobacco products) has been suggested as a way to reduce the risks of tobacco smoking. This trial is designed to investigate whether changes in toxicant exposure after switching from a commercial to reduced toxicant prototype (RTP) cigarette (7 mg International Organisation for Standardisation (ISO) tar yield) can be assessed by measurement of biomarkers and other factors. The primary objective is to descriptively assess changes in selected biomarkers of exposure (BoE) and biomarkers of biological effect (BoBE) within participants and within and between groups after switching. Secondary objectives are to assess similarly changes in other biomarkers, quality of life, smoking behaviours, physiological measures, mouth-level exposure to toxicants and sensory perception.

**Methods/design:**

This trial will assess current smokers, ex-smokers and never-smokers in a single-centre single-blind, controlled clinical trial with a forced-switching design and in-clinic (residential) and ambulatory (non-residential) periods. Smokers will be aged 23–55 years (minimum legal smoking age plus 5 years) and non-smokers 28–55 years (minimum legal smoking age plus 5 years, plus minimum 5 years since last smoked). Smokers will be allowed to smoke freely at all times. We will assess changes in selected BoE and BoBE and effective dose in urine and blood after switching. Creatinine concentrations in serum, creatinine clearance in urine, cotinine concentration in saliva, diaries and collection of spent cigarette filters will be used to assess compliance with the study protocol. Mouth-level exposure to toxins will be assessed by filter analysis.

**Discussion:**

Data from this study are expected to improve scientific understanding of the effects of RTP cigarettes on BoE and BoBE, and give insights into study design for clinical assessment of potential MRTPs.

**Trial registration:**

The study was registered in the Current Controlled Trials database under the reference ISRCTN81286286.

## Background

Despite universal acceptance that smoking is harmful, a substantial number of adults continue to smoke. There is likely to continue to be a substantial population of smokers for the foreseeable future (1.6 billion smokers worldwide is predicted in the year 2040) even with the current tobacco control measures [1]. The concept of tobacco harm reduction, defined by the US Institute of Medicine (IOM) as “decreasing total morbidity and mortality, without completely eliminating tobacco and nicotine use” [2] is being considered by some regulators.

### Development of potential reduced-exposure products

In the 2001 IOM report, *Clearing the Smoke: the scientific basis for tobacco harm reduction*[2], the development of potential reduced-exposure products (PREPs) was suggested as a possible way to achieve tobacco harm reduction. A PREP was defined as a product that results in substantial reduction in exposure to one or more tobacco toxicants and that can reasonably be expected to reduce the risk of developing one or more specific diseases or other adverse health effects. The types of studies, including clinical trials, required to assess PREPs are being considered [3]. In 2011 the IOM issued a further report on the science needed to assess modified risk tobacco products (MRTPs) in response to the US FDA who have released draft guidance on this.

In addition, a scientific committee that advises the World Health Organization (WHO) has recommended the mandated reduction of certain cigarette smoke toxicants [4]. The potential for toxicant exposure and risk reduction may be tested by the development and assessment of reduced toxicant prototype (RTP) cigarettes. An RTP has been developed with the use of novel technologies related to tobacco, filter, and cigarette format which have been employed to reduce the yields of smoke toxicants. The tobacco used in the RTP has been selected for smoking acceptability and chemistry profiles (e.g., low in tobacco-specific nitrosamines and heavy metals) and a proportion of the blend prepared with a new process where tobacco is extracted with water and the extract and fibres are treated separately to reduce certain toxicant precursors before being recombined [5]. Additionally, an inert non-tobacco sheet that contains a high percentage of glycerol is also cut and mixed with the tobacco to dilute the concentrations of smoke toxicants during smoking [6]. In order to reduce the yields of some vapour phase toxicants, filters in the RTP incorporated a highly adsorbent form of carbon [7] and a selective amine-functionalised resin [8].

### Previous clinical study assessments

The tools to assess exposure to smoke toxicants were examined in two previous clinical studies conducted in Germany and Canada using conventional cigarettes. These studies aimed to assess whether the available techniques could adequately assess human smoke exposure by filter analysis and measurement of biomarkers of exposure (BoE) [9-11]. These enable the exposure tobacco smoke toxicants or their metabolites to be measured in urine, or other biologic matrices. Both studies showed a good correlation between exposure estimates from the two methods, and, in the case of the German study, the methods were senstive enough to detect changes in smoke exposure following a switch to a cigarette with a lower International Organization for Standardization (ISO) tar yield [10].

Technologies used in the RTPs have previously been shown to significantly reduce yields of a range of tobacco smoke toxicants by 10% to 95% compared with typical commercial products with equivalent ISO tar yields [12]. A clinical study of these RTPs, using a short-term (6-week) switching design, determined smoke toxicant exposure by measurement of various BoE in current smokers after they switched from conventional cigarettes to an RTP (ISRCTN 72157335) [13]. This study showed that toxicant exposure was reduced after switching to the RTP, although the prototypes required further development to optimise the reductions and improve acceptability to smokers.

However, reductions in smoke toxicant exposure alone should not be assumed to result in a reduction in smoking-related risk, and the studies noted above did not investigate the potential for a reduction in risk of smoking-related diseases or adverse biological effects. Biomarkers of biological effect (BoBE) may serve as biological indicators of the body’s response to smoke exposure. These include modifications in some parameters of blood composition, alterations in activity of specific enzymes, the appearance of DNA adducts, localised increases in messenger RNA and protein concentrations, and the appearance of specific antibodies (autoantibodies). BoBE may differ between smokers, non-smokers and ex-smokers [3,14]. Ideally, smoking-related biomarkers should be detectable early and have plausible links to the early stages of smoking-related diseases. While reductions in exposure to toxicants can be seen within 2–4 weeks by measurement of BoE, it is anticipated that changes in biological effect measurable by changes in BoBE, physiological or quality-of-life assessments would take longer to manifest. Hatsukami *et al.*[3] recommended that a series of clinical studies of both short and intermediate duration be performed in the assessment of PREPs/MRTPs. Previous studies (unpublished data) indicate that a period of 6 months should be sufficient to demonstrate the effects of reductions in exposure.

Three biomarkers have been identified that are suggested in the literature to have the potential to enable assessment of participant compliance [15,16]. These biomarkers, termed biomarkers of effective dose (BoED), are DNA and protein adducts of identified smoke toxicants that have relatively long half-lives (50–55 days). Interrogation of these data, therefore, could be useful for the monitoring of behaviour and compliance over extended periods of time.

Other elements to consider in clinical studies are mouth-level exposure (MLE) to tar and nicotine [13] and whether changes in exposure affect any basic physiological features or quality of life. Sensory features are also important to assess, particularly in relation to compliance to protocol in a switching study.

### Study hypothesis and objectives

The study is intended to test the hypothesis that switching of smokers from conventional cigarettes to RTP cigarettes will result in measurable reductions in exposure to toxicant and lead to beneficial changes in biological effects, as shown by assessment of biomarkers.

The primary objective of this study is to descriptively assess changes in selected BoE and BoBE (Table 1), within participants and within and between groups, after a forced switch from a commercial control cigarette to an RTP cigarette of equivalent ISO tar yield. Secondary objectives are to descriptively assess changes within participants and within and between groups in further BoE, BoBE and BoED, quality of life, smoking behaviours, physiological measures, MLE and sensory perception, and to descriptively assess changes in the primary and secondary end points together after switching, in comparison with values for ex-smokers and never-smokers (Table 1). Data from this study are expected to improve the scientific understanding of tobacco products.

**Table 1:** Parameters in primary and secondary endpoints

**Primary endpoints**	
***Biomarkers of exposure***	
	• Urine: Nicotine, cotinine, 3-hydroxycotinine and their glucuronide conjugates, acrolein metabolite (3-HPMA), crotonaldehyde metabolite (HMPMA), TSNA metabolites (total NNAL, total NNN, total NAT, total NAB), 1,3-butadiene metabolite (MHBMA), acrylonitrile metabolite (CEMA) and aromatic amines (4-aminobiphenyl, 3-aminobiphenyl, o-toluidine, 2-aminonaphthalene), plus 9 PAH metabolites (1-Hydroxynapthalene, 2-Hydroxynapthalene, 2-Hydroxyflourene, 1-, 2-, 3-, 4, and 9-Hydroxyphenanthrene, 1-Hydroxypyrene)
	• Salivary cotinine
	• Exhaled CO
***Biomarkers of biological effect:***	
	• Urine: F_2_-isoprostane (8-iso-PGF2 Type III and VI; for oxidative stress)
	• Blood: white blood cells (for cardiovascular disease)
	• Plasma: sICAM-1 (for cardiovascular disease)
**Secondary endpoints**	
***Biomarkers of exposure***	
	• Urine: urine mutagenicity (Ames test)
***Biomarkers of biological effect***	
	• Urine: 8-OHdG, cis-thymidine glycol, 11-dehydrothromboxane B2
	• Blood: gene expression, neutrophil count, monocyte count
	• Erythrocytes: haemoglobin, superoxide dismutase activity, glutathione peroxidase, glutathione reductase, catalase activity, malondialdehyde
	• Plasma or serum: IL-6, IL-8, ascorbic Acid, dehydroascorbic Acid, total antioxidant capacity, hsCRP (males), fasting (12 h) lipid profile (total cholesterol, LDL, HDL, Triglycerides), fibrinogen, MCP-1, neutrophil elastase, MMP-1, MMP-9, TIMP-1, LTB4, TNF-α, VEGF, oxLDL
***Other features***	
	• Filter analysis: MLE to nicotine and ‘tar’ (nicotine-free dry particulate matter [NFDPM])
	• Sensory testing questionnaire
	• QoL questionnaire
	• Smoking behaviours questionnaire
	• Puffing and inhalation behaviour
	• Pulmonary function test (FEV, FEV1, PEF)
	• Biomarkers of Effective Dose (compliance monitoring)
	• Haemoglobin adducts in blood: Acrylonitrile (2-Cyanoethylvaline) and 4-ABP

*3-HPMA*, 3-Hydroxypropylmercapturic acid; *HMPMA*, 3-hydroxy-1-methylpropylmercapturic acid; *TSNA*, Tobacco-specific nitrosamine; *NNAL*, 4-(methylnitrosamino)-1-(2-pyridyl)-1-butanol; *NNN*, *N*-nitrosonornicotine; *NAT*, *N*-nitrosoanatabine; *NAB*, *N*-nitrosoanabasine; *MHBMA*, monohydroxybutenylmercapturic acid; *CEMA*, 2-cyanoethylmercapturic acid; *PAH*, Polyaromatic hydrocarbons; *CO*, Carbon monoxide; *PGF2*, prostaglandin F2; *sICAM-1*, soluble intercellular adhesion molecule; *8-OHdG*, 8-oxo-2′-deoxyguanosine; *IL-6*, Interleukin 6; *IL-8*, Interleukin 8; *hsCRO*, high-sensitivity C-reactive protein; *LDL*, Low-density lipoprotein; *HDL*, High-density lipoprotein; *MCP-1*, Monocyte chemotactic protein-1; *MMP-1*, Matrix metalloproteinase-1; *MMP-9*, Matrix metalloproteinase-9; *TIMP-1*, Tissue inhibitor of metalloproteinase 1; *LTB4*, Leukotriene B4; *TNF-α*, Tumour necrosis factor-alpha; *VEGF*, Vascular endothelial growth factor; *oxLDL*, oxidised low-density lipoprotein; *MLE*, mouth level exposure; *NFDPM*, nicotine free dry particulate matter; *QoL*, Quality of life; *FEV*, Forced expiratory volume; *FEV1*, Forced expiratory volume in 1 second; *PEF*, Peak expiratory flow; *4-ABP*, 4-aminobiphenyl.

## Methods/design

### Study design

This study will be a single-centre, single-blind, controlled clinical trial with a forced-switching design that will be conducted in Hamburg, Germany. The study will be partly clinic-based (residential) and partly ambulatory (non-residential, including brief visits to the clinic). This study will be conducted in compliance with the ethical principles of the Declaration of Helsinki, Good Clinical Practice [17,18] and German law. When the Principal Investigator signs the protocol, he or she will be agreeing to adhere to the instructions and procedures described in it.

The Principal Investigator or designated investigators will ensure that participants are given full and adequate oral and written information in non-technical terms about the nature, purpose, potential risks and possible benefits of study participation. Participants will be given time to consider all the information, the opportunity to ask questions and will be required to read, sign and date informed consent forms that summarise the discussion before participating in any procedures related to the study.

The protocol and the informed consent forms have been approved by the ethics committee of the Ärztekammer Hamburg, Hamburg, Germany (Processing Number PV3824).

The study has a forced-switching design to enable data to be obtained about changes in exposure to tobacco smoke toxicants and related biological effects. The effects of RTP cigarettes will thus be evaluated within individuals (switching smokers serve as their own control) and between individuals (switching versus non-switching smokers). The ex-smoker and never-smoker control groups will provide background levels of BoE arising from environmental sources. They will also provide reference levels of BoBE that would be expected in people who have given up smoking for at least 5 years or who have never smoked. The inclusion of multiple types of non-smoker control groups is anticipated to improve the interpretation of the data generated in this study.

Financial compensation for the inconvenience to and effort of participants will be offered as part of the study, but the sponsor does not wish this approach to incentivise participants to smoke. Stipends will, therefore, be calculated independently by the clinic according to the usual rates for this type of clinical study and will be approved by the ethics committee of the Ärztekammer Hamburg.

### Study participants

#### Identification of study participants

Participants will be recruited into the study through advertisements on the clinic’s own website and local advertising that will not refer to the characteristics of the study products. All study participants (smokers and non-smokers) will be sourced from the Hamburg area from the same communities. Adult, healthy participants of either sex and of any ethnic origin will be enrolled. The planned ratio of male to female participants within each subgroup of the smoking groups is between 3:2 and 2:3, that is, an attempt will be made to enrol approximately equivalent numbers of male and female subjects into each subgroup, within these tolerances.

#### Inclusion criteria

The suitability of participants who give informed consent will be assessed according to the inclusion criteria within 28 days before entering the study and verified upon arrival at the clinic for the first in-clinic evaluation period. The universal inclusion criteria are weight of at least 52 kg (men) or 45 kg (women) and body-mass index within the normal range; no relevant clinically abnormal findings on physical examination, electrocardiography, clinical laboratory testing or lung function tests, or in the medical history, as judged by the investigators; the ability to communicate well with the investigators and understand and comply with the requirements of the study; willingness to refrain from consuming alcohol within 72 h before the first day of each in-clinic evaluation (an alcohol breath test result of up to 0.05% alcohol at the start of a visit might be tolerated, at the discretion of the investigators); willingness to refrain from consuming and avoid being in the presence of the cooking of grilled, fried or barbequed food for 48 h before the first day of each in-clinic evaluation; not being pregnant or breastfeeding and using a reliable method of contraception (e.g., abstinence or barrier methods with spermicide from at least one menstrual cycle before the first day of the study until at least one menstrual cycle after the last in-clinic evaluation, surgical sterilsation of self or partner ≥6 months before the study, an intrauterine device in place or hormonal contraceptives started ≥3 months before the study until at least one menstrual cycle after the last in-clinic evaluation), or being postmenopausal with amenorrhea for at least 2 years.

Additional inclusion criteria for the smoking groups are age 23–55 years; being a regular current smoker of a brand of cigarettes with an ISO tar yield of 6–8 mg and a blend style and mechanics similar to brands sold in Germany; having smoked the same brand for a minimum of 6 months and any brand for at least 5 years before screening; smoking of 10–30 cigarettes per day; willingness to switch to an RTP cigarette and to smoke only products provided during the study; and having a urinary cotinine level of >100 ng/mL.

Additional inclusion criteria for the ex-smoking group are age 28–55 years; not having smoked for at least 5 years but having been a regular smoker of 10–30 cigarettes per day for at least 5 years previously; having a urinary cotinine level of <30 ng/mL (inclusive of levels 0 and 1 on the NicAlert™ test, Palico, Rotkreuz, Switzerland) at screening and <100 ng/mL (inclusive of levels 0–2 on the NicAlert™ test) during the study. Additional inclusion criteria for the never-smoking group are age 28–55 years; never having smoked more than 100 cigarettes during his or her lifetime, and none in the previous 5 years; and having a urinary cotinine level of <30 ng/mL (inclusive of levels 0 and 1 on the NicAlert™ test) at Screening and <100 ng/mL (inclusive of levels 0–2 on the NicAlert™ test) during the study.

Documented exceptions to the inclusion criteria may be permitted at the discretion of the investigators, in agreement with the sponsor, providing risks to participants would not be increased and the realisation of the scientific objectives of the study would not be hindered. Occasional use of paracetamol (up to 4 g per day) might be permitted at the discretion of investigators. Should the need arise to take any other medications, investigators should be informed as soon as possible; if safety allows, the need should be reported before medication is started. All medication administered during the study will be documented, either by the participant during ambulatory periods or by investigators during in-clinic evaluations. In the case of other medical complaints, participants should inform the physician in charge of the study. A physician will be on duty during the entire course of the study, although participants will be referred to their personal physician whenever appropriate (e.g., for the treatment of chronic medical conditions).

#### Exclusion criteria

Exclusion criteria may be applied at screening or at any time during the study. The universal exclusion criteria are clinically relevant gastrointestinal, renal, hepatic, neurological, haematological, endocrine, oncological, urological, pulmonary, immunological, psychiatric or cardiovascular disorders or any other conditions that, in the opinion of the investigators, would jeopardise the safety of the participant or affect the validity of the study results; abnormal findings on physical examination, in the medical history, or in clinical laboratory results deemed clinically relevant by investigators; participation in a previous clinical trial within 30 days before entering the study; donation or loss of at least 400 mL blood within 90 days before entering the study; donation of plasma within 7 days before entering the study; acute illness (e.g., upper-respiratory-tract infection, viral infection etc.) requiring treatment within 4 weeks before entering the study; regular use of any nicotine or tobacco products other than commercially manufactured filter cigarettes; self-reported non-inhaling smoking behaviour (drawing of smoke into the mouth and throat without inhaling); observed as being non-inhalers by the clinic staff at first in-clinic evaluation; history of drug or alcohol abuse within 24 months before screening; positive alcohol breath test and/or urine screen for illicit drugs; positive result for HIV or hepatitis; use of prescription or over-the-counter bronchodilator medications (e.g., inhaled or oral β-agonists) to treat chronic conditions within 12 months before entering the study; use of any medications that interfere with the cyclo-oxygenase pathway (e.g., aspirin or ibuprofen) within 14 days before entering the study; use of any prescribed systemic medication within 14 days before entering the study (except for hormonal contraceptives and hormone-replacement therapy); use of any drugs or substances (except tobacco) known to be strong inducers or inhibitors of CYP (cytochrome P450) enzymes within 28 days before entering the study (Table 2); strenuous physical activity (exceeding the participant’s normal activity levels) within 7 days before screening or in-clinic evaluations; pregnancy; and employment or being first-degree relatives to employees of the tobacco, journalism (television and radio), public relations, market research or advertising industries or the clinic.

**Table 2:** **Strong inducers or inhibitors of CYP (cytochrome P450) enzymes**[19]

**INHIBITORS**			
**1A2**	**2B6**	**2C8**	**2C19**
Cimetidine	Thiotepa	Gemfibrozil	Fluoxetine
Fluoroquinolones	Ticlopidine	Montelukast	Fluvoxamine
Fluvoxamine			Ketoconazole
Ticlopidine			Lansoprazole
			Omeprazole
			Ticlopidine
**INHIBITORS**			
**2C9**	**2D6**	**2E1**	**3A4,5,7**
Amiodarone	Amiodarone	Disulfiram	HIV antivirals:
Fluconazole	Bupropion		Indinavir
Isoniazid	Chlorpheniramine		Nelfinavir
	Cimetidine		Ritonavir
	Clomipramine		Amiodarone
	Duloxetine		NOT azithromycin
	Fluoxetine		Cimetidine
	Haloperidol		Clarithromycin
	Methadone		Diltiazem
	Mibefradil		Erythromycin
	Paroxetine		Fluvoxamine
	Quinidine		Itraconazole
	Ritonavir		Ketoconazole
	Doxepin		Mibefradil
			Nefazodone
			Troleandomycin
			Verapamil
**INDUCERS**			
**1A2**	**2B6**	**2C8**	**2C19**
N/A	Phenobarbital	N/A	N/A
	Phenytoin		
	Rifampin		
**2C9**	**2D6**	**2E1**	**3A4,5,7**
Rifampin	N/A	Isoniazid	Carbamazepine
Secobarbital			Phenobarbital
			Phenytoin
			Pioglitazone
			Rifabutin
			Rifampin
			St John’s wort
			Troglitazone

(Adapted with permission from Version 5.0 (http://medicine.iupui.edu/clinpharm/ddis/table.aspx)).

1A2, 2B6 etc = CYP enzyme subtypes.

For the smoking groups an additional exclusion criterion at screening is planning to quit smoking in the next 12 months (although cessation counselling will be available to smoking participants during and at the end of the study). For the non-smoking groups, additional exclusion criteria are smoking at any time during the study and regular exposure to second-hand smoke (e.g., through living in a household or working with smokers).

#### Withdrawal from the study

Participants will be allowed to withdraw from the study at any time. If they do so, they will receive a pro rata stipend. Investigators may at any time withdraw a participant from the study if they deem this action to be in his or her best interest or that of other participants. The end-of-study examination should be undertaken when a participant leaves the study, if he or she agrees, and reasons for withdrawal should be recorded. If patients cannot be assessed because he or she cannot be contacted after non-attendance of a scheduled event, they will be reported as lost to follow-up.

Participants will be informed of the importance of conforming to all requirements of the protocol, but violations might not result in removal from the study, as the sponsor realises that 100% compliance for every participant over the 6 month period of the study is unlikely. Honesty about protocol violations will, therefore, be encouraged. Participants will be asked to record and report at each clinic visit during ambulatory periods instances when they have not adhered to the protocol, or at check in for in-clinic evaluations. Investigators will decide whether non-compliant participants may remain in the study. Although withdrawals or dropouts are not expected to be replaced, the Sponsor will consider replacing participants to maintain the power of the study.

### Study groups

**Figure 1:**
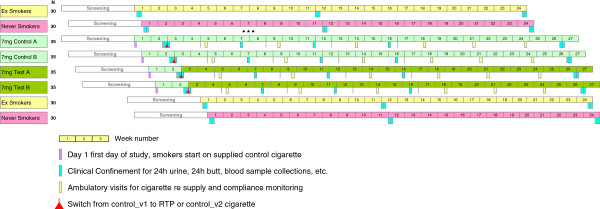
Study design and scheduled events.

Participants will be assigned to study groups primarily on the basis of whether they are smokers, ex-smokers or never smokers. The demographics for each group will be matched as far as possible (accepting the constraints of the minimum age difference between smokers and non-smokers) i.e. participants’ sex and age will also be taken into account. The assignment of control cigarettes or RTP cigarettes will be kept within cohorts, i.e., all participants in a smoker subgroup will be switched to the same study product at the same time to meet the restrictions posed by the predetermined schedule for the conduct of the study. For practical reasons, including clinic capacity and sample collection, the smoker and non-smoker groups will be further split into two subgroups (Figure 1). Therefore, to ensure enough participants are recruited to all subgroups, that subject availability will be assured and groups will be well matched for age and gender, we have accepted that full randomisation is not possible. Smokers will be recruited and subgroups filled according to order of screening, age, gender and availability at the study start time. Non-smokers will be recruited on the basis of the same criteria in an attempt to match the control group well with the smoker-group demographics.

The study events will be scheduled to avoid as far as possible overlap of subgroups for in-clinic evaluations; non-smoking subgroups will never be resident in the clinic at the same time as any smoking subgroup.

### Investigational products

All study cigarettes will be provided by the sponsor free of charge (Table 3). To achieve blinding, unbranded cigarettes and packs will be manufactured specifically for the study. All packaging will include the mandatory health warnings, details of yields and tax stamps. The control cigarettes will match the specifications of the commercial cigarettes supplied before switching, except for a visual but cosmetic difference (i.e., plain white tipping on the control versus cork patterned tipping on the commercial cigarette; Table 3). This will allow RTP and control smoker cohorts to see a noticeable change to their cigarettes without being able to determine which product they are receiving. The different cigarette products will be identifiable to the clinic pharmacy staff and investigators by study identification codes (Table 3) that will be printed on the packaging and on the individual cigarettes. The cigarette products will be stored in a locked, limited-access area under controlled temperature and humidity conditions.

**Table 3:** Investigational products

**Study period**	**Control group**	**Test group**
Days 1–14	Lucky Strike Silver (ID W861) with mono CA filter, cork-print tipping and commercial tobacco blend	Lucky Strike Silver (ID W861) with mono CA filter, cork-print tipping and commercial tobacco blend
	*Yields using ISO methodology: 7 mg tar, 0.6 mg nicotine, 8 mg CO*	*Yields using ISO methodology: 7 mg tar, 0.6 mg nicotine, 8 mg CO*
Days 15–183	Lucky Strike Silver (ID H285) with mono CA filter, white tipping and commercial tobacco blend	RTP (ID G429) with triple CA/CR20/charcoal filter^a^, white tipping and study tobacco blend (20% non-tobacco sheet, 50% treated tobacco and 30% commercial blend).
	*Yields using ISO methodology: 7 mg tar, 0.6 mg nicotine, 8 mg CO*	*Yields using ISO methodology: 7 mg tar, 0.7 mg nicotine, 8 mg CO*^*a*^

*ID,* study identification code, *CA,* cellulose acetate, *CR20,* amine-functionalised resin filter additive, *CO,* carbon monoxide, *RTP,* reduced-toxicant prototype cigarette, *ISO,* International Organisation for Standardisation. ^a^to be confirmed.

### Study procedures at each visit

#### Screening

Participants who have signed informed consent forms will be eligible for screening. Medical history, smoking history and demographic data (e.g., sex, age, ethnic origin, etc.) will be recorded. Each participant will be required to provide proof of age with a form of identification accepted by MOMENTUM Pharma Services (i.e., national identity card or work or residence permit).

Participants will undergo the following assessments: physical examination, including measurement of height, weight, blood pressure, pulse rate and temperature; 12-lead electrocardiography; pulmonary function testing; clinical laboratory tests; testing for infection with hepatitis virus B and C and HIV; urine pregnancy test for women; urine drug screening; and alcohol breath test. Urine will be taken for cotinine testing to confirm whether participants are smokers or non-smokers. Other general observations and questioning may be added by the investigators.

#### Ambulatory visits

Participants in the smoking groups will visit the clinic on the first day of the first ambulatory period (Figure 1). Smoking subjects will be asked to confirm the ISO tar yield of their usual brand and smoking status of all subjects will be verified by a urine cotinine test. Participants will be given a diet sheet to which they will be asked to adhere during specified times in ambulatory periods (i.e. 48 h before each in-clinic evaluation). Participants will be supplied with cigarettes according to their assigned product (Table 3) and daily consumption rate, plus two packs, and asked to smoke only the supplied brand until their next visit. Smokers will be allowed to smoke *ad libitum*, rather than according to a pre-specified quantity or timetable.

Each participant will be trained how to use an electronic diary in which he or she will be asked to document cigarette consumption, diet, exercise, medications and any other health-related issues on every ambulatory day. Participants will be required to bring the diary to every clinic visit.

Further ambulatory clinic visits will be made on days 31, 62, 95, 124, 144 and 165 (Figure 1). During these visits, diary entries will be checked, any protocol violations (e.g. consumption of cigarettes that were not supplied by the sponsor, significant health-related events or intake of excluded medications) will be assessed and decisions made about further participation in the study. Urine samples will be taken and tested for cotinine. Cigarette butts will be collected for analysis and the numbers of butts will be compared with diary entries and recorded. Participants will be supplied with further study cigarettes (normal daily consumption x number of days, plus two packs) and reminded to smoke only those until their next visit. They will also receive fresh tins to collect cigarette butts. The clinic staff will record information on hard copy case report forms during visits and will later be transcribed into an electronic system.

#### In-clinic evaluations

The first in-clinic evaluation for the smoking groups will be on days 12–15 (Figure 1, Table 4). Participants will check in on the evening of the first day and remain in the clinic until the evening of the final day. Participants will be instructed at check in on how to request cigarettes and return the butts within the clinic. During this and all other in-clinic evaluations, sufficient amounts of cigarettes for each smoker per day, transferred in participant-specific containers, will be made available by the site pharmacy and the clinic pharmacy staff and investigators will keep dispensing records. All cigarettes will be smoked only in a designated smoking area with appropriate ventilation.

**Table 4:** Schedule for first in-clinic evaluation of smokers

**Day and suggested times**	**Sampling**	**Procedure**
**12**		
1700 h (check in)	Blood, urine	Measure BP, PR, RR and temperature; abbreviated physical examination and medical history; illicit drug, alcohol and cotinine testing; pregnancy testing; collection of diary and cigarette butts; dinner
1900 h	None	Start first 24 h urine collection, monitoring of cigarette consumption and collection of filters
2100 h	None	Snack
**13**		
0700 h	Blood	Measure BP, PR, RR and temperature; blood collection for creatinine in serum, biomarkers and gene expression
0800 h	None	Breakfast
To be scheduled	None	Assess smoking behaviour^a^
1000 h	None	Administer sensory, quality-of-life and diet and lifestyle questionnaires^b^
1200 h	None	Lunch
1500 h	None	Measure exhaled CO
1700 h	Saliva	Saliva cotinine testing; dinner
1900 h	None	End first and start second 24 h urine collection, monitoring of cigarette consumption and collection of filters
2100 h	None	Snack
**14**		
0700 h	Blood	Measure BP, PR, RR and temperature; blood collection for creatinine in serum, biomarkers and gene expression
0800 h	None	Breakfast
To be scheduled	None	Assess smoking behaviour^a^
1100 h	None	Pulmonary-function test
1200 h	None	Lunch
1500 h	None	Measure exhaled CO
1700 h	Saliva	Saliva cotinine testing; dinner
1900 h	None	End second 24 h urine collection, monitoring of cigarette consumption and collection of filters; switch to next assigned product
2100 h	None	Snack
**15**		
0700 h	None	Measure BP, PR, RR and temperature
0800 h	None	Breakfast
1000 h	None	Administer sensory questionnaire
1200 h	None	Lunch
1700 h	None	Dinner
1900 h	None	End filter collection; dispense interventional products and cigarette butt collection tins for use at home, remind participants of instructions for diet and completion of diaries

*BP,* blood pressure, *PR,* pulse rate, *RR,* respiratory rate *CO,* carbon monoxide, ^a^To be performed by sponsor. ^b^May be done on day 14.

On the evening of day 14, after completion of the 24 h urine collection, smokers will be switched to their next assigned product (control or RTP). Upon discharge on the morning of Day 15, participants will be given sufficient supplies of the new product and butt collection tins to last until the next visit. After switching, smokers will undergo four more in-clinic evaluations on days 45–46, 76–77, 108–109 and 181–183 (Tables 5 and 6).

**Table 5:** Schedules for second, third and fourth in-clinic evaluations of smokers

**Days and suggested times**	**Sampling**	**Procedure**
**45, 76, 108**		
1700 h (check in)	Blood, urine	Measure BP, PR, RR and temperature; abbreviated physical examination and medical history; illicit drug, alcohol and cotinine testing; pregnancy testing; collection of diary and cigarette butts; clinical laboratory tests; electrocardiography; lung- function tests; dinner
1900 h	None	Start 24 h urine collection, monitoring of cigarette consumption and collection of filters
2100 h	None	Snack
**46, 77, 109**		
0700 h	Blood	Measure BP, PR, RR and temperature; measure creatinine in serum; biomarkers and gene expression
0800 h	None	Breakfast
1000 h	None	Administer sensory questionnaire; administer quality-of-life and diet and lifestyle questionnaires^a^
1200 h	None	Lunch
1500 h	None	Measure exhaled CO
1700 h	Saliva	Cotinine testing; dinner
1900 h	None	End 24 h urine collection, monitoring of cigarette consumption and collection of filters; dispense interventional products and cigarette butt collection tins for use at home, remind participants of instructions for diet and completion of diaries

*BP,* blood pressure, *PR,* pulse rate, *RR,* respiratory rate, *CO,* carbon monoxide, ^a^On day 108–109 visit only; may be conducted on either day.

**Table 6:** Schedule for final in-clinic evaluation of smokers

**Day and suggested times**	**Sampling**	**Procedure**
***181***		
1700 h (check in)	Blood, urine	Measure BP, PR, RR and temperature; abbreviated physical examination and medical history; illicit drug, alcohol and cotinine testing; pregnancy testing; collection of diary and cigarette butts; dinner
1900 h	None	Start first 24 h urine collection, monitoring of cigarette consumption and collection of filters
2100 h	None	Snack
**182**		
0700 h	Blood	Measure BP, PR, RR and temperature; measure creatinine in serum; biomarkers and gene expression
0800 h	None	Breakfast
To be scheduled	None	Assess smoking behaviour^a^
1000 h	None	Administer sensory, quality-of-life and diet and lifestyle questionnaires†
1200 h	None	Lunch
1500 h	None	Measure exhaled CO
1700 h	Saliva	Cotinine testing; dinner
1900 h	None	End first and start second 24 h urine collection, monitoring of cigarette consumption and collection of filters
2100 h	None	Snack
**183**		
Anytime (check out)	None	Measure BP, PR, RR and temperature; physical examination; electrocardiography
0700 h	Blood	Measure creatinine in serum; biomarkers
0800 h	None	Breakfast
To be scheduled	None	Assess smoking behaviour^a^
1100 h	None	Pulmonary-function test
1200 h	None	Lunch
1500 h	None	Measure exhaled CO
1700 h	Saliva	Cotinine testing; dinner
1900 h	None	End second 24 h urine collection, monitoring of cigarette consumption and collection of filters; dispense adequate commercial products of normal ISO tar yield for 7 post-study days

*BP,* blood pressure, *PR,* pulse rate, *RR,* respiratory rate, *CO,* carbon monoxide, ^**a**^To be performed by sponsor. †May be done on day 183.

In previous smoking studies, most smokers (>90%) have increased the number of cigarettes they smoke on the last day of the study, typically by two to three cigarettes per day, which can adversely affect statistical outcomes. This increase is thought to be due to the impending end of the supply of free cigarettes. To mitigate this effect, at discharge from the last in-clinic evaluation, all smoking participants will be supplied with 7 days’ worth of their baseline product and asked to record daily consumption over the next 7 days. Participants will be contacted by telephone on the 7th day to obtain data on consumption.

In previous smoking studies based at the Hamburg clinic, it has been an absolute requirement of the ethics committee that smoking cessation counselling is provided to smoking subjects during the study. Therefore, several optional smoking-cessation workshops will be held during the study, for groups of 20–25 participants and led by a psychologist experienced in smoking cessation. The format will be a mix of lectures (including presentations, demonstrations and case studies), self-assessment (tests and questionnaires) and team and small-group work. The main aims of workshops will be to improve the knowledge of the participants about smoking cessation options, help participants to make realistic self-assessments regarding smoking cessation, increase participants’ motivation to quit or reduce smoking and develop individual plans for quitting or reducing smoking. Each workshop will take 60–90 min. Additionally, the Smoke-Free Programme of the Munich Institut für Gesundheitsförderung will be offered to all smoking subjects who wish to participate. The approach involves three meetings of 180 min and two telephone consultations. Following a preparatory phase, a day to quit smoking will be decided for all attendees. Strategies to handle irritation and critical situations and for behaviour control will be provided and alternative behaviours developed. In the final phase, the emphasis will be on prevention of relapse. Participants will be reminded of the dangers of smoking before enrolment, will be free to voluntarily quit smoking and withdraw from the study at any time and may still access the smoking-cessation workshops.

Ex-smokers and never smokers will be assessed in three in-clinic evaluations, on days 1–3, 78–80 and 162–164 (Figure 1, Table 7). As for the smoking groups, they will arrive in the evening of the first day and will remain in the clinic until the evening of final day of each visit.

**Table 7:** Schedule for in-clinic evaluations of ex-smoker and never-smoker groups

**Day and suggested times**	**Sampling**	**Procedure**
**1, 78, 162**		
1700 h (check in)	Blood, urine	Measure BP, PR, RR and temperature; abbreviated physical examination and medical history; illicit drug, alcohol and cotinine testing; pregnancy testing; dinner
1900 h	None	Start first 24 h urine collection
2100 h	None	Snack
**2, 79, 163**		
0700 h	Blood	Measure BP, PR, RR and temperature; measure creatinine in serum; biomarkers and gene expression^a^
0800 h	None	Breakfast
1000 h	None	Administer quality of life and diet and lifestyle questionnaires^b^
1200 h	None	Lunch
1500 h	None	Measure exhaled CO
1700 h	Saliva	Cotinine testing; dinner
1900 h	None	End first and start second 24 h urine collection
2100 h	None	Snack
**3, 80, 164**		
0700 h	Blood	Measure creatinine in serum; biomarkers
0800 h	None	Breakfast
1000 h	None	Administer quality of life and
1100 h	None	Pulmonary-function test§
1200 h	None	Lunch
1500 h	None	Measure exhaled CO
1700 h	Saliva	Cotinine testing; dinner
1900 h	None	End second 24 h urine collection
**164 (check out)**		
Any time	Blood, urine	Measure BP, PR, RR and temperature; physical examination; electrocardiography; clinical laboratory tests

^a^Not on day 79. ^b^Not during visit on days 1–3; may be performed on day 79 or 80 and on day 163 or 164.

### Assessments

#### Cigarette consumption

Participants will be required to document cigarette consumption during ambulatory periods in diaries. In addition, on selected days, participants will be asked to collect the butts of smoked cigarettes in specially supplied tins and bring them to the next clinic visit. Butts will be counted to confirm the accuracy of diary entries on consumption. Participants will also be required to present any un-smoked cigarettes and the empty packets from smoked cigarettes. During in-clinic evaluations smokers’ cigarette consumption will be monitored by the clinic staff. Cigarettes will be issued to participants by staff one at a time, upon request, as they enter the designated smoking area and the butts of all smoked cigarettes will be collected by staff when smokers leave the smoking area.

#### Clinical and bioanalytical laboratory tests

The tests shown in Table 8 will be performed at screening for smokers and non-smokers. All tests, except for the HIV and hepatitis serology, will also be performed during the smokers’ in-clinic evaluation on days 108–109 and at the end of the study.

**Table 8:** Clinical laboratory tests

**Haematology**	
●	Haemoglobin
●	Haematocrit
●	Total and differential leukocyte count (neutrophils, eosinophils, basophils, monocytes, lymphocytes)
●	Red-blood-cell count
●	Platelet count
●	Mean corpuscular volume
●	Mean corpuscular haemoglobin
●	Mean corpuscular haemoglobin concentration
**Clinical chemistry**	
●	Blood urea nitrogen
●	Creatinine^a^
●	Total bilirubin
●	Alkaline phosphatase
●	AST
●	ALT
●	Sodium
●	Potassium
●	Glucose (fasting)
●	Calcium
●	Chloride
●	Creatinine kinase
●	γ-glutamyl transpeptidase
●	Total protein
●	Uric acid
●	Urea
**Urinalysis (by sticks as part of clinical laboratory investigation)**	
●	pH
●	Specific gravity
●	Protein
●	Glucose
●	Ketones
●	Bilirubin
●	Blood
●	Nitrite
●	Urobilinogen
●	Leukocytes
●	**Additional tests**
●	Alcohol breath test^b^
●	Urine cotinine^b^
●	HIV
●	Hepatitis surface antigen
●	Hepatitis C virus
●	Urine drug screening^b^
	○ Amphetamines
	○ Barbiturates
	○ Benzodiazepines
	○ Cocaine
	○ MDMA
	○ Methamphetine
	○ Morphine
	○ Methadone
	○ Tricyclic antidepressants
	○ Tetrahydrocannabinol
●	Urine pregnancy test^b^

All tests, except the additional tests, will also be performed during the smokers’ day 108–109 in-clinic evaluations and at the end of the study.

^a^To be repeated in each 24 h in-clinic evaluation for all participants.

^b^To be repeated at all in-clinic evaluation visits for smokers and non-smokers.

##### Urine tests

Before the start of each 24 h urine collection period during in-clinic evaluations (Tables 5, 6, 7), each participant will be required to empty his or her bladder. Following this, each void will be collected in separate labelled brown plastic bottles and stored at 2-8°C. Collected urine will be pooled and stored at 2–8°C, labelled with the day on which collection ended. The total volume of urine collected over each 24 h period will be calculated from weight and specific gravity. After thorough mixing, samples will be transferred to labelled tubes and stored at −18 to −25°C. The details of biomarker assessments are shown in Table 9.

**Table 9:** Urinary biomarkers

**Biomarker**	**Number and volume of aliquots**
4-Aminobiphenyl, 3-Aminobiphenyl,	2 aliquots of 12 mL each
o-Toluidine, 2-Aminonaphthalene	
OH-PAHs suite	2 aliquots of 12 mL each
Nicotine + 5	2 aliquots of 6 mL each
3-HPMA, HMPMA, CEMA	2 aliquots of 3 mL each
MHBMA	2 aliquots of 3 mL each
Total NNAL, NNN, NAB and NAT	2 aliquots of 10 mL each
F_2_-isoprostane (8-iso-PGF2 Type III)	2 aliquots of 6 mL each
F_2_-isoprostane (8-iso-PGF2 Type VI)	2 aliquots of 6 mL each
11-dehydrothromboxane B2	2 aliquots of 3 mL each
Urine mutagenicity	1 aliquots of 300 mL each
8-OHdG	2 aliquots of 1.5 mL each
Cis-thymidine glycol	2 aliquots of 1.5 mL each

*OH-PAH*, phenolic polycyclic aromatic hydrocarbons.*3-HPMA*, 3-Hydroxypropylmercapturic acid.

*HMPMA*, 3-hydroxy-1-methylpropylmercapturic acid. *CEMA*, 2-cyanoethylmercapturic acid.

*MHBMA*, monohydroxy-3-butenylmercapturic acid. *NNAL*, 4-(methylnitrosamino-1-(3-pyridyl)-1-butanol.

*NNN*, N-nitrosonornicotine. *NAB*, N-nitrosoanabasine. *NAT*, N-nitrosoanatabine.

*8-OhdG*, 8-Oxo-2′-deoxyguanosine.

Three haemoglobin adducts of identified smoke toxicants will be measured as BoED. Additionally, BoE that can yield information on short-term compliance, for example assessment of the ratio of a BoE for a smoke toxicant that does not change significantly between the products (such as nicotine) to a smoke toxicant that does significantly change (such as acrolein or NNK), will also be measured.

To ensure that every urine collection is complete for all participants, 24 h creatinine clearance will be calculated during the 24 h urine collection period. Creatinine clearance values will be verified by investigators, taking into account urine volumes and serum creatinine results. Values outside the normal clinical range (1.33–1.92 mL/s/m^2^) may be classified as acceptable, since values have been reported to vary greatly between individuals and a substantial proportion are likely not to fall within this range.

Samples available for long-term storage after the study will be kept at the sponsor’s biological sample archive.

##### Saliva tests

Saliva samples will be collected with Salivettes without citric acid (Sarstedt, Nuembrecht, Germany) at specified times during in-clinic evaluations (Tables 4, 5, 6, 7). The cellulose dams will be gently moved around the mouth for 2 min and light chewing will be allowed. The loaded dams will then be returned to the original tubes before centrifugation at 1000 *g* for 2–5 min at room temperature. The clear saliva sample will be stored at −20°C until assay. Cotinine concentrations will be measured with a validated assay (Celerion, Lincoln, NE, USA).

##### Blood tests

Blood samples will be collected in blood-collection tubes. The blood volumes required for biomarker analyses and the biomarkers to be assessed are detailed in Table 10. Global gene expression profiles will be analysed with GeneChip expression arrays (Affymetrix, Santa Clara, CA, USA; [20]), which are accepted as an industry standard [21] and have been used successfully for clinical studies [22]. Any unused split samples will be transferred to the sponsor’s biological samples archive at the end of the study, at least until publication of the study results, to allow for any necessary repeat analyses.

**Table 10:** Biomarkers in blood

**Biomarker**	**Collection tube volume and type**	**Matrix**
2-cyanoethylvaline	2 × 4.9 mL EDTA	Erythrocytes
4-ABP Hb adduct	2 × 4.9 mL EDTA	Erythrocytes
IL-6, IL-8, MCP-1, TNF-a, VEGF, MMP-1, MMP-9, TIMP-1, ox LDL, superoxide dismutase activity, glutathione peroxidase activity, glutathione reductase activity, catalase activity, Hb (lysate)	1 × 9 mL EDTA	Plasma, lysate
Total antioxidant capacity	1 × 4.7 mL serum	Serum
Ascorbic acid, dehydroascorbic acid	1 × 7.5 mL EDTA	Plasma
Neutrophil elastase, fibrinogen	1 × 5 mL citrate	Plasma
Hb (lysate), malondialdehyde	1 × 7.5 mL li-heparin	Lysate, cell pellet
sICAM-1	1 × 4 mL EDTA	Plasma
hsCRP (males)	1 × 8.5 mL serum	Serum
Lipid Profile	1 × 8.5 mL serum	Serum
LTB4	1 × 4 mL li-heparin	Plasma
White blood cells, neutrophils, monocytes, haemoglobin	1 × 2.7 mL EDTA	Plasma
Gene expression	2.5 mL × 2 Paxgene	Plasma

*4-ABP Hb adduct*, 4-aminobiphenyl haemoglobin adduct. *IL-6*, interleukin-6. *IL-8*, interleukin-8.

*MCP-1*, Monocyte Chemoattractant Protein 1. *TNF-a*, Tumor necrosis factor alpha.

*VEGF*, Vascular Endothelial Growth Factor. *MMP*, Matrix Metalloproteinase.

*TIMP-1*, Tissue inhibitor of metalloproteinases. ox *LDL*, oxidised low density lipoprotein.

*Hb*, Haemoglobin. *sICAM-1*, soluble intercellular adhesion molecule. *hsCRP*, high sensitivity C-reactive protein.

*LTB4*, Leukotriene B4. *EDTA*, Ethylenediaminetetraacetic acid.

#### Exhaled carbon dioxide

Expired carbon monoxide (CO) levels will be measured with the EC50 Micro III Smokerlizer CO meter (Bedfont, Maidstone, UK), or similar device at specified times (Tables 4, 5, 6, 7).

#### Mouth-level exposure to nicotine and tar

MLE to nicotine and tar will be estimated by a method developed and published by the sponsor [23]. A portion is cut from the mouth end of used cigarette filters collected from all cigarettes smoked over a 24 h period. The sponsor will provide the clinic with airtight collection tins for each participant, labelled with his or her identification code, for use during each in-clinic evaluation. Collected cigarette filters will be stored at room temperature until dispatch to the sponsor for MLE analysis (usually within 1 week).

At the sponsor’s laboratories, the filter tips will be placed in solvent and the extract will be analysed for nicotine content and ultraviolet absorbance to show the tar content. The data will be compared with calibration curves, obtained from machine-smoking regimes where smoke nicotine and tar yields are plotted against tip nicotine and ultraviolet absorbance values; the slopes of the curves represent the filtration efficiency of the cigarette filter. These filtration efficiency data and the amount of nicotine and tar retained on the cigarette filter will enable estimation of the amount of smoke that exited the filter and entered the smokers’ mouths during puffing (MLE).

#### Puffing, inhalation and exhalation measurements

Significant changes in smokers puffing and/or inhalation/exhalation behaviour following the product switch could affect the primary outcomes of this study. Therefore, puffing behaviour will be assessed in a normal smoking environment with the devices Smoking Analyser version 7 (SA7) and breathe in/breathe out (BIBO) apparatus, both of which have been designed, developed and produced by the sponsor and are described elsewhere [24]. The SA7 uses pressure transducers, built into a hand-held cigarette holder, to measure puff flow, volume and duration whilst the BIBO uses similar transducers in a second hand-held unit to measure inhalation/exhalation parameters. Both devices connect to a common-interface box and laptop computer where puffing and inhalation/exhalation behaviour is recorded.

Two cigarettes will be smoked per measurement through holders on the SA7 and BIBO apparatus. After each puff, drawn through the SA7 holder, participants will be asked to inhale and exhale up to five times through the BIBO. They will wear nose clips during the measurements but must try to puff, inhale and exhale as normally as possible. This system provides a complete record of the subject’s smoking behaviour, including puff volume, puff duration, number of puffs, total puff volume, mouth-hold duration, depth and time of inhalation and exhalation time.

Tests will be performed before and after switching for smokers in the switching groups and at similar time points for smokers in the non-switching groups. Owing to this study design allowing smokers to smoke when they wish, however, the specific timings of these measurements cannot be scheduled *a priori*, but attempts will be made to make the timings as close as possible on each measurement day for individual participants. The butts from the cigarettes smoked in these tests will be pooled with the others collected for the subject that day and sent to the sponsor for analysis.

#### Pulmonary function

Forced expiratory flow, forced expiratory flow in 1 s and peak expiratory flow will be measured at screening and once at the last in-clinic evaluation visit for every participant. Additional measurements will be obtained once in an in-clinic evaluation before switching and once during an in-clinic evaluation after switching in the smoking groups only.

#### Quality-of-life, sensory and diet and lifestyle assessments

Participants will be asked to self-administer study-specific questionnaires, written in German, on a tablet device during the in-clinic evaluation visits.

A prototype quality-of-life questionnaire, developed by an independent expert and funded by the sponsor , will be administered before switching and at the end of the study in the smoking groups, and in the middle and at the end of the study for the non-smoking groups. It will capture multi-dimensional data on individuals’ subjective perceptions on their daily lives, physical, psychological and social functioning, and well-being.

A second questionnaire will be administered to smoking groups before and multiple times after switching to assess participants’ sensory impression of the products and possibly assist in the interpretation of the toxicant exposure and compliance data. Questions take into account standard sensory attributes associated with cigarette smoking, including acceptability, draw effort, amount of smoke, satisfaction, irritation, nicotine impact, taste amount and quality, mouth dryness and aftertaste. Each attribute will be scored as magnitude and liking.

Aspects of an individual’s diet and lifestyle could affect the biomarkers under investigation in this study. Therefore, a third questionnaire will be administered to smoking and non-smoking groups and aims to provide an estimate of the participants’ normal standards of diet and lifestyle.

### Statistical analysis

The sample size estimate is based on observed data for BoE and BoBE and calculations made with MINITAB software version 15 (two-sample *t*-test). Percentages of reductions in smoke toxicants in the RTP versus commercial products are assumed to translate into corresponding reductions in BoE concentrations. Sample size calculations were performed to achieve a power of 80% to detect a difference between control and test products at an alpha level of 0.05. On the basis of the least sensitive BoE (aromatic amines), a sample size of 50 was determined to be adequate for all BoE of interest in this study (from study ISRCTN 72157335).

Similar estimates for BoBE are based on data for biomarkers in urine and plasma previously obtained by the sponsor and the least sensitive biomarkers in the literature, which showed that sample size of 50 would be adequate for BoBE in the primary objective. Literature search for secondary BoBE responses were uninformative, so sample size was assumed to be the same as for the primary outcomes.

On the basis of these calculations and a 6-week study in groups of 50 smokers (ISRCTN 72157335), it was estimated for this study that 70 participants per smoking group will be required to allow for anticipated attrition rates of up to 16% and achieve completion by at least 50 participants in each group. Sixty participants will be recruited to each of the ex-smokers and never-smoker control groups, as attrition is expected to be lower in these groups.

Statistical analyses will be performed by Celerion. A detailed statistical analysis plan will be prepared before database closure and agreed by the sponsor and Celerion. Any changes in the planned statistical methods will be documented in the study report.

The primary and secondary objectives will be examined by computing group biomarker levels at baseline, midpoints and at the end of the study (Table 1). Data for the primary outcome might be transformed to ensure that any assumptions associated with particular statistical tests or models are obeyed. ANOVA will be performed to identify differences between groups. As cigarette consumption may change over time or as a result of the switch, an ANCOVA will also be performed, using cigarettes per day as a covariate. For the secondary outcomes, an exploratory analysis with a matrix-type approach, such as principle component analysis, may be used.

**Figure 2:**
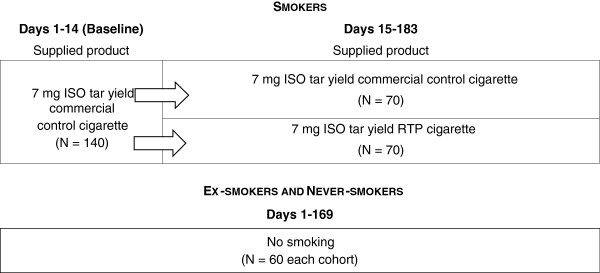
Participant groups, group size and product switching.

A diagram summarising participant groups, group size and product switching is shown as Figure 2

## Discussion

A previous clinical study demonstrated that switching smokers to prototype cigarettes with reduced levels of certain toxicants in the smoke results in a reduction of corresponding biomarkers of exposure [13]. This exposure reduction cannot be extrapolated to a reduction in risk. Therefore, this study has been designed to investigate whether longer term use of such products leads to continued exposure reduction and whether there is any change in the levels of biomarkers of biological effect (BoBE). Advantageous changes in BoBE may assist in a “weight of evidence” assessment of the potential for these products to reduce risk.

Data from this study are expected to increase scientific understanding of combustible tobacco products with reduced toxicant yields and of how biomarkers may be used to assess short-term and long-term effects in the context of exposure and risk. The results will be published in peer-reviewed scientific journals.

## Abbreviations

BoE: Biomarker of exposure; BoBE: Biomarker of biological effect; BoED: Biomarker of effective dose; BIBO: Breath in/breath out apparatus; FDA: Food and Drug Administration; IOM: Institute of Medicine; ISO: International Organisation for Standardisation; ISRCTN: International Standard Randomised Controlled Trial Number; MLE: Mouth level exposure; MRTP: Modified risk tobacco product; PREP: Potential reduced exposure prototype; RTP: Reduced toxicant prototype; SA7: Smoking analyser version 7; WHO: World Health Organisation.

## Competing interests

CJS, NN and AE are current employees of British American Tobacco and the work was to be funded by British American Tobacco. DG is currently employed by Celerion who were to be involved in project and data management of the study. IM is the principal investigator at the clinic where the study is to be conducted and is employed by Momentum Pharma Services in Hamburg, Germany.

## Authors’ contributions

All authors contributed to the development of the study protocol, and co-wrote the protocol and manuscript. All authors read and approved the final manuscript.

## Pre-publication history

The pre-publication history for this paper can be accessed here:

http://www.biomedcentral.com/1471-2458/13/690/prepub
